# *Bacillus licheniformis* normalize the ileum microbiota of chickens infected with necrotic enteritis

**DOI:** 10.1038/s41598-018-20059-z

**Published:** 2018-01-29

**Authors:** Shuai Xu, Yicen Lin, Dong Zeng, Mengjia Zhou, Yan Zeng, Hesong Wang, Yi Zhou, Hui Zhu, Kangcheng Pan, Bo Jing, Xueqin Ni

**Affiliations:** 1 Animal Microecology Institute, College of Veterinary Medicine, Sichuan Agricultural University, Sichuan Province Chengdu, 611130 China; 2 Key Laboratory of Animal Disease and Human Health of Sichuan Province, Sichuan Agricultural University, Sichuan Province Chengdu, 611130 China

## Abstract

Necrotic enteritis (NE) is a severe intestinal disease, which can change gut microbiota and result in a high cost for the poultry industry worldwide. However, little is known regarding how the gut microbiota of NE chicken ileum are changed by *Bacillus licheniformis*. This study was conducted to investigate how ileum microbiota structure was changed by *B. licheniformis* in broiler chickens challenged with *Clostridium perfringens*-induced NE through Illumina MiSeq sequencing. The broilers were randomly separated into four groups: the negative control group (NC), the positive control group (PC), the fishmeal and coccidia group (FC), and the PC group supplied with feed containing *B. licheniformis* (BL). Compared to the PC and FC, alpha diversity, beta diversity, and the bacterial taxa of the ileum microbiota were more similar in BL and NC. Some genera, which were related to the NE control, became insignificant in BL with NC, such as *Lactobacillus*, *Lactococcus*, *Bacteroides*, *Ruminococcus* and *Helicobacter*. The PICRUSt analysis revealed that a tumour suppressor gene, p53, which was negatively correlated with *Helicobacter*, was enriched in the BL group. Our findings showed that the ileum microbiota disorder caused by NE in chickens was normalized by dietary *B. licheniformis* supplementation.

## Introduction

Necrotic enteritis (NE) in chickens, which was first reported by Parish in 1961^1^, is a common illness caused by *Clostridium perfringens*^2^. There is increasing evidence that NE outbreaks in broiler chickens have a severe economic impact and globally cost over $2 billion annually in losses and disease control because of the high mortality rates and reduced growth performance^3^. NE not only causes economic losses but also results in illnesses in humans. NE in chickens can cause a threat to public health through the food chain by *C. perfringens*^4^. *C. perfringens* is a strictly anaerobic gram-positive bacterium, which can form spores. *C. perfringens* commonly presents in the intestinal tract of chickens but is not pathogenic and causes enterotoxaemia only under certain conditions. An experimental disease challenge trial showed that it is necessary to introduce induction factors, such as *Eimeria* co-infection and high protein feed supplementation (like fishmeal), to cause diseases^5,6^. Research on NE in chickens has been conducted for decades, but it is still one of the major challenges in the poultry industry. This is especially true since in-feed antibiotics have been banned in more and more countries^7^, and it is increasingly important to search for alternatives for the treatment of NE in chickens.

Probiotics are “friendly” bacteria that help to maintain a normal balance in the intestinal tract by aiding normal digestion, supporting the immune system and promoting overall health^8^. They can likely prevent and treat disease effectively by mainly modulating mucosal immune activity and epithelial barrier function as a biological antagonist, which has already been proven through clinical trials for maintaining disease treatment^9^. In addition, probiotics are characterized as safe, free from pollution and have no remaining compounds. Probiotics have become the ideal alternative for antibiotics. *Bacillus subtilis* and *Lactic acid bacteria* are common probiotics, and *Bacillus licheniformis*, which is “generally recognized as safe”, has been extensively used for a long time in the poultry industry^3^. *B. licheniformis* has promising affects in many industries, such as poultry and fishing, as a supplement. Research showed that supplementing feed water with *B. licheniformis* could enhance the growth performance of chickens^10^. This mechanism may explain how *B. licheniformis* could produce different enzymes, such as lipase, protease and amylase, which could increase the digestion and absorption ability of chickens^11^. Meanwhile, the metabolite of *B. licheniformis* could also help prevent some diseases, such as piglet diarrhoea and NE in chickens^12^.

The chicken’s ileum is an important site for digestion and nutrient absorption^13^ and is home to a large and varied microbiota community^14^. Several studies have shown that NE could induce disease of the intestines and change the intestinal microbiota structure of patients, especially for the ileum^15,16^. Chicken intestinal physiology and health were influenced by these changes. Therefore, the objective of this study was to investigate how the ileum microbiota structure was changed by *B. licheniformis* in broiler chickens challenged with *C. perfringens*-induced NE through high-throughput next-generation sequencing.

## Results

### Metadata and sequencing

A total of 60 chicken ileum samples (15 NC, 15 PC, 15 FC and 15 BL) were collected and sent for sequencing. After selecting the OTU and checking for chimaera, a total of 1,532,759 reads were assigned to 4,883 non-singleton OTUs from 60 samples. Each sample had 25,546 ± 9,653 reads and 428 ± 172 OTUs on average (Table S1). We used a Venn diagram, which is based on the OTUs, to compare the similarities and differences between the microbiota community of the different groups. Compared with NC, the FC, PC and BL communities had 1483, 1359 and 1205 shared OTUs (Figure S1), with the OTUs comprising 50.43%, 43.87% and 49.32% of the sequences in the FC, PC and BL communities, respectively.

### The microbiota diversity analysis

**Figure 1:**
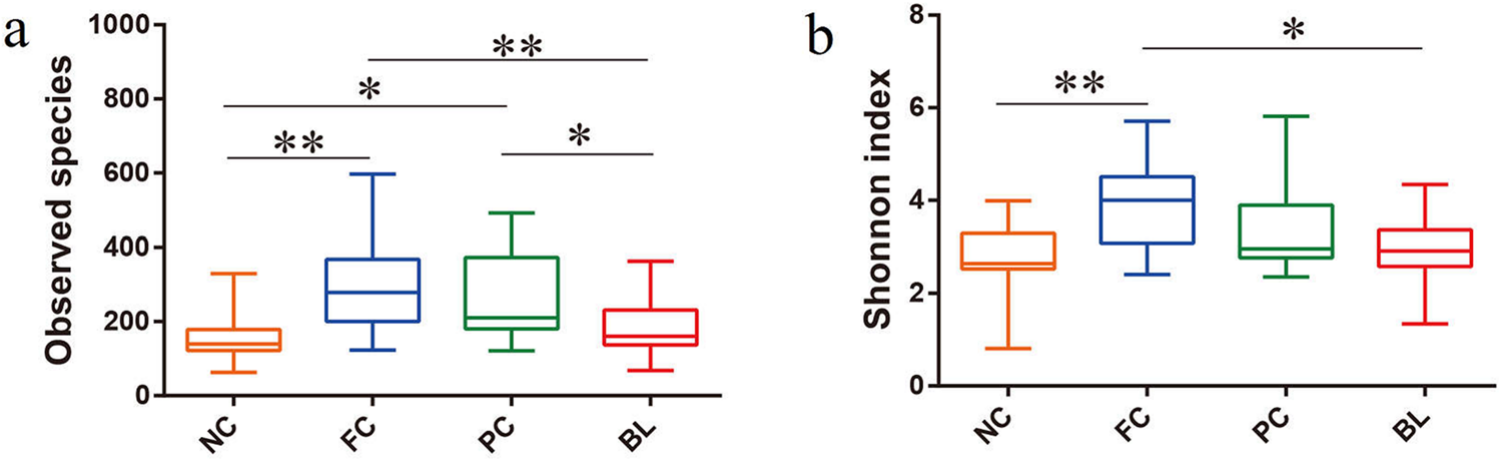
Alpha diversity analysis of the ileum gut microbiota of NC, FC, PC and BL. Panel a represents differences in bacterial community richness (Observed species) between the four groups. Panel b represents differences in bacterial community diversity (Shannon) between the four groups. Asterisk shows significant differences between groups (**P < 0.01, *P < 0.05, Mann-Whitney U test).

The alpha diversity analysis was included for the observed species and the Shannon index, which was intended to be representative of the community richness and community diversity. Figure 1 shows the quartile deviation of observed species and the Shannon index. Noticeably, the community richness and community diversity in chickens with NC and BL were lower than those of PC and FC by the observed species (Fig. 1a) and the Shannon index (Fig. 1b). We found that BL and NC were significantly different from FC (*P* < 0.01) and were significantly different from PC (*P* < 0.05) in the observed species by boxplot. For the community diversity comparison, samples of NC were significantly different from those of FC (*P* < 0.01), and those of the BL group were also significantly different from those of FC (*P* < 0.05) in the Shannon index. However, no significant difference in Alpha diversity analysis was observed between the samples of NC and BL (*P* > 0.05).

**Figure 2:**
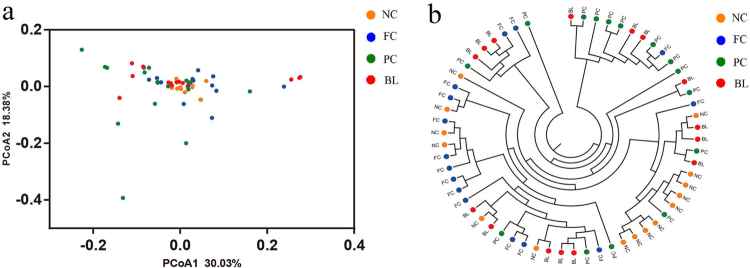
Principal Coordinate Analysis (PCoA) and Gut microbiota trees of Unifrac distances of the ileum gut microbiota of NC, FC, PC and BL. Panel a represents a 2 dimensional weighted PCoA plot by sample type. Panel b represents the Gut microbiota trees clustering on weighted Unifrac distances. Sequences were normalized to the depth of 9,718 sequences with 10 times of subsampling to minimize the effect of sequencing depth.

The principal coordinates analysis (PCoA) and the gut microbiota trees, which revealed the similarity measure of bacterial communities based on the phylogenetic distance, were performed based on the weighted Unifrac distance matrixes (Fig. 2). The results of PCoA showed a degree of diversity discrepancy between each group, but the samples from NC, BL and FC clustered together, which was consistent with the results of the gut microbiota tree.

### Comparison of microbiota in the ileum in each group

**Figure 3:**
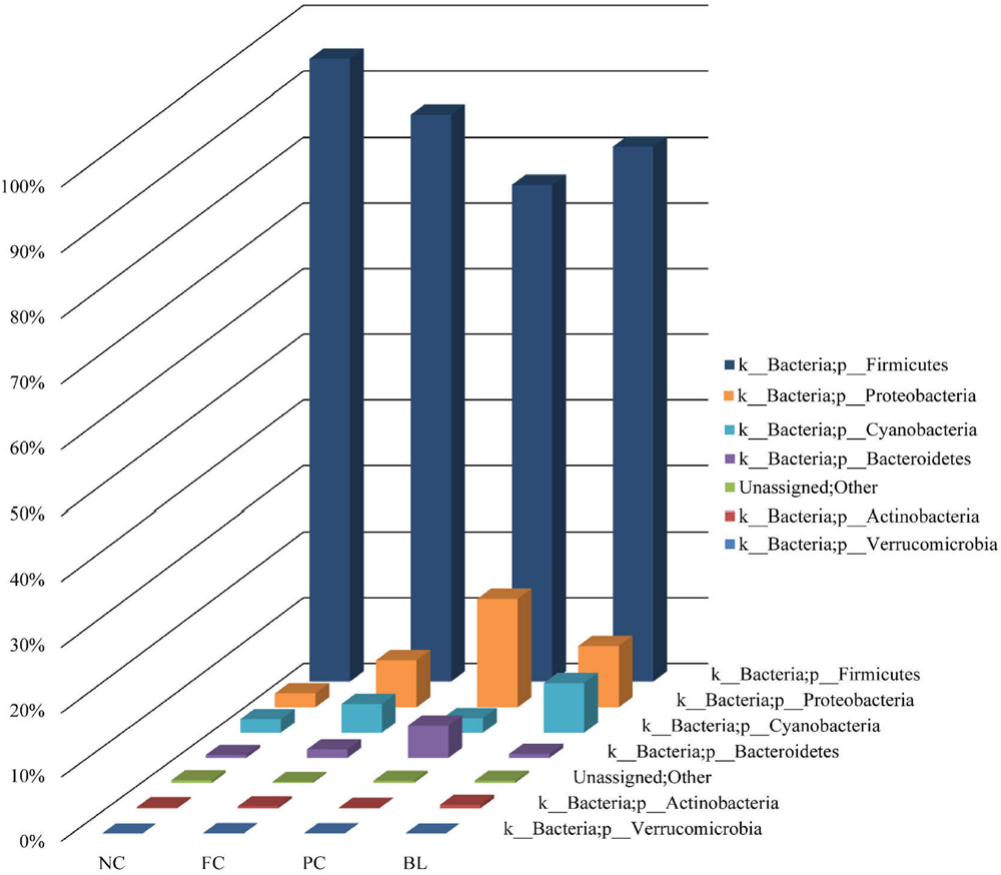
Relative abundance of the broilers’ ileum microbiota in level phylum. The abundances of each groups are more than 0.01%.

At the phylum level, the abundance, whose relative abundance was more than 0.1% in all of the groups, were classified into 7 phyla, and 6 were identified as a phylum, and the average relative abundances of them were over 99.50% of the overall bacteria community. Firmicutes, Proteobacteria and Bacteroidetes were significantly different in NC and PC. Proteobacteria and Bacteroidetes increased with the severity of necrotic enteritis (NC, FC and PC), whereas Firmicutes decreased. However, this phenomenon we called microbiota disorder was alleviated in BL group (Fig. 3).

**Figure 4:**
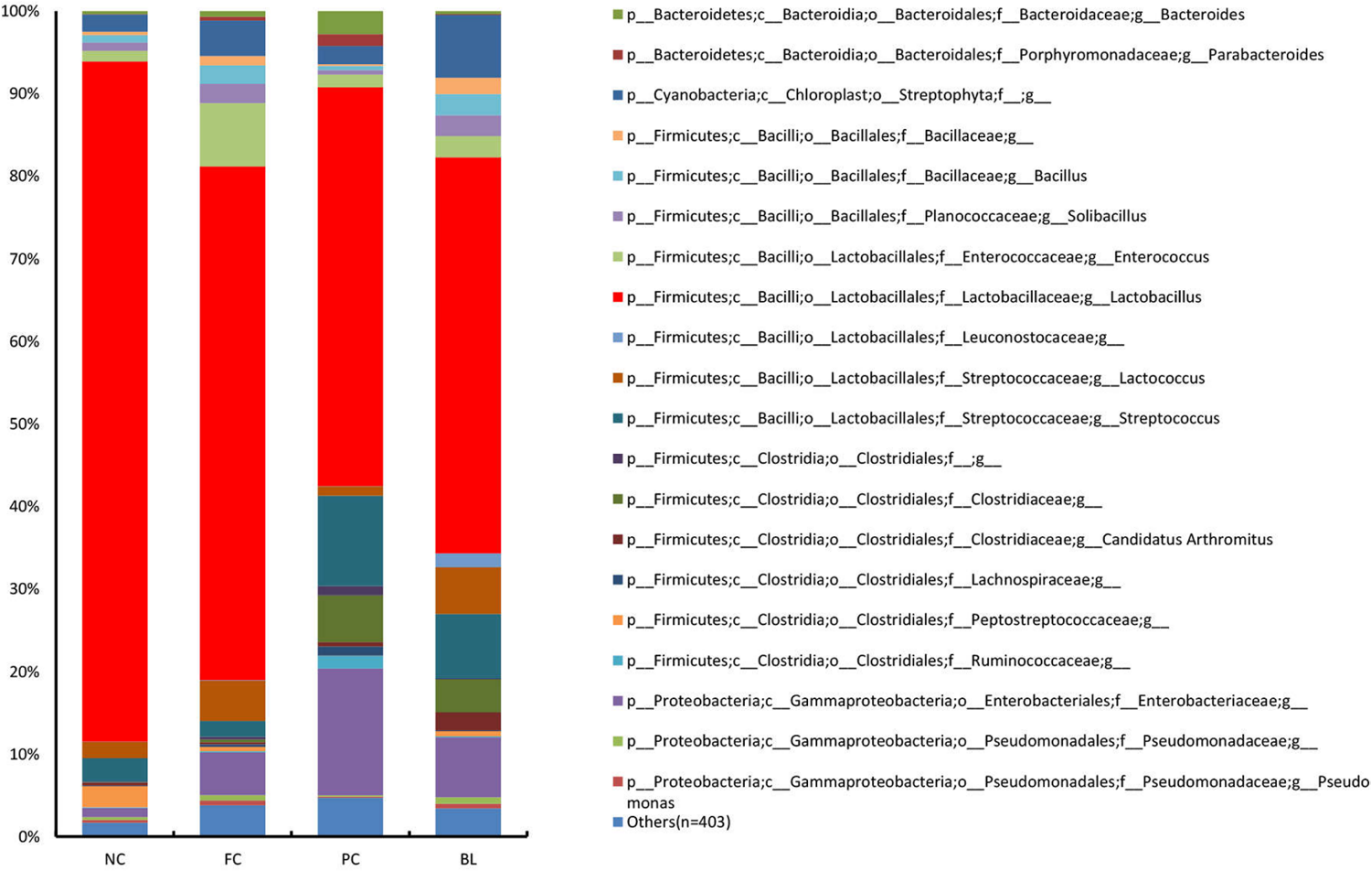
Microbiota composition of NC, FC, PC and BL. Each bar represents the average relative abundance of each bacterial taxon within a group. The top 20 abundant taxa are shown.

**Figure 5:**
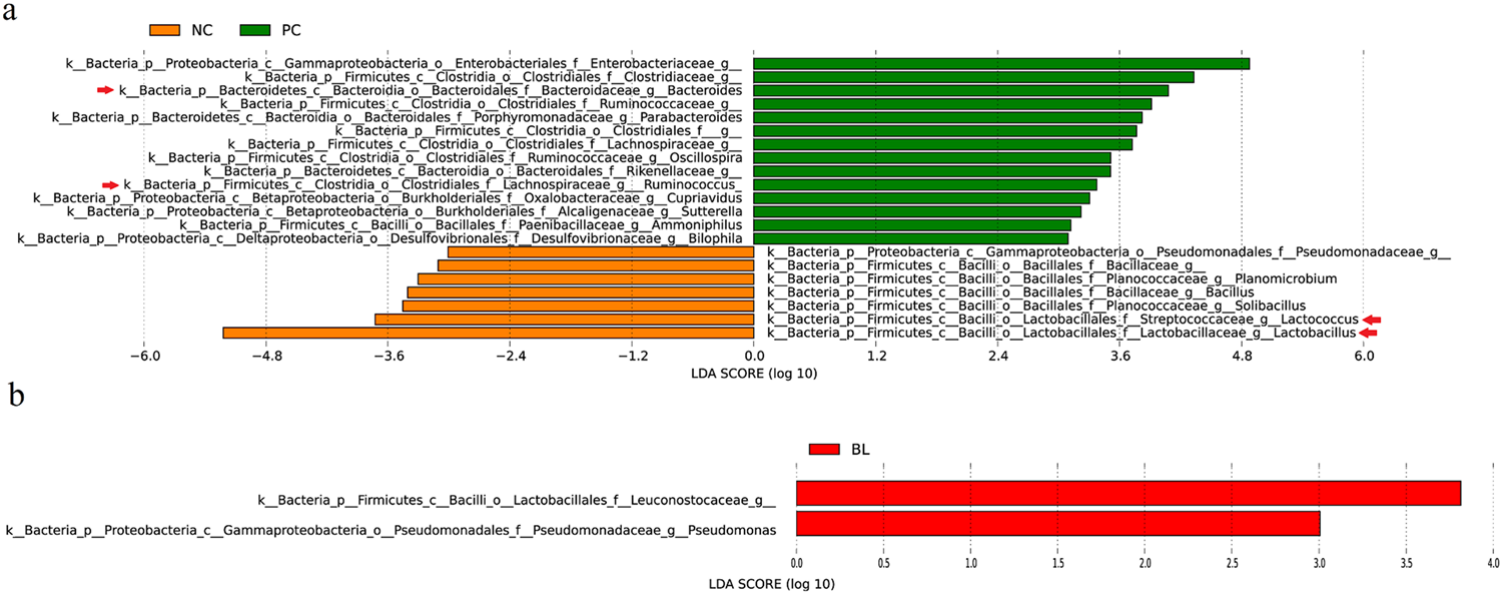
Taxa that were significantly differentially represented between groups were examined by linear discriminant analysis coupled with effect size (LEfSe) using the default parameters (LDA score = 3). Panel a show different taxa between NC and PC. Panel b show different taxa between NC and BL. The mean and median relative abundance of these bacterial taxa are indicated with straight and dotted lines, respectively.

**Figure 6:**
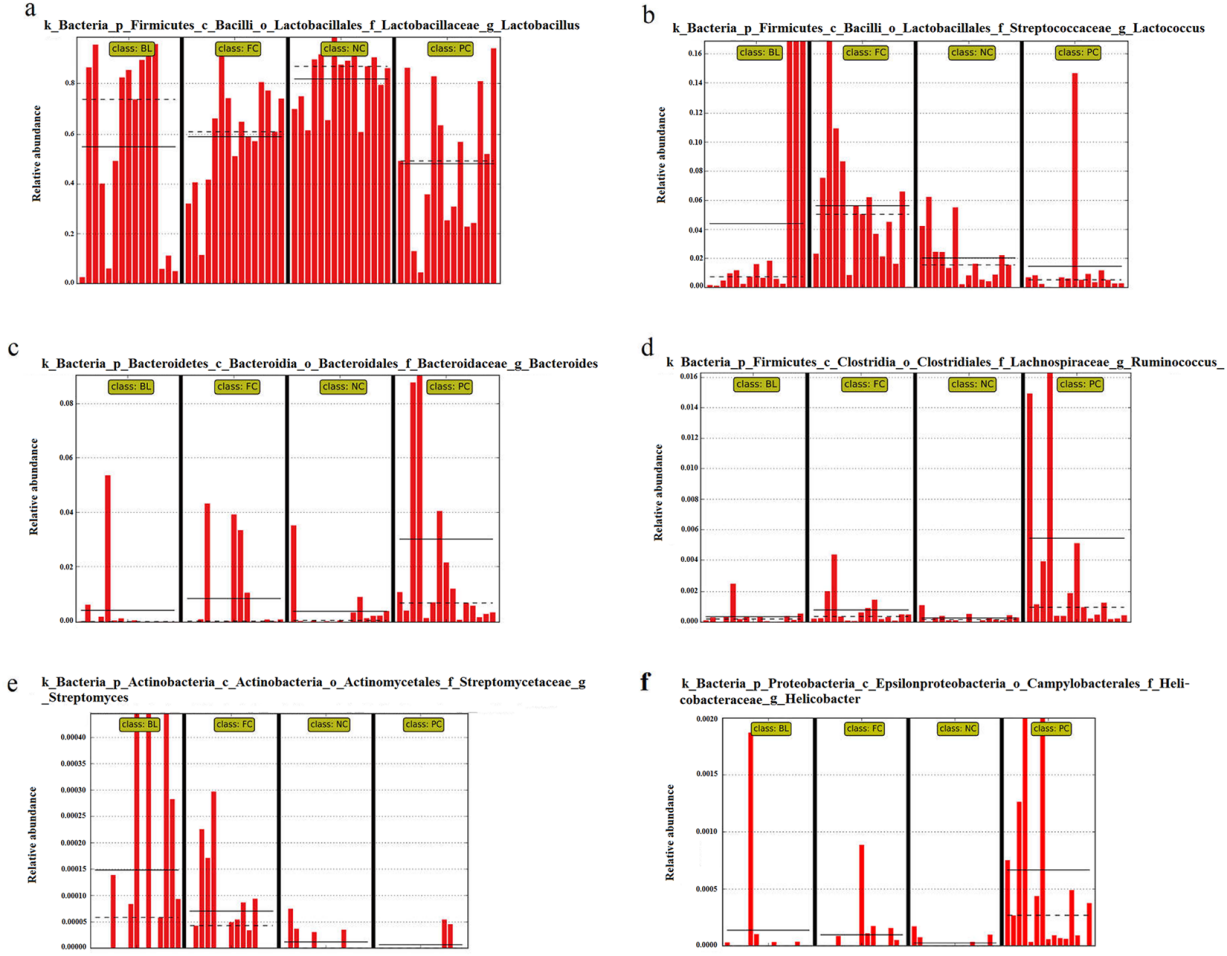
The relative abundance of 6 bacterial taxa in 4 groups (NC, FC, PC and BL). Panel a show relative abundance of *Lactobacillus*. Panel b show relative abundance of *Lactococcus*. Panel c show relative abundance of *Bacteroides*. Panel d show relative abundance of *Ruminococcus*. Panel e show relative abundance of *Streptomyces*. Panel f show relative abundance of *Helicobacter*.

Figure 4 shows the structures of the bacterial community of four groups in the chicken ileum samples. At the genus level, 422 genera were detected. The top 20 genuses with higher relative abundance, of which the average relative abundance was over 95.31% of the overall bacteria community, are illustrated. We performed LEfSe (LDA score = 3) to identify significant differences in the bacterial taxa. This threshold could guarantee that the meaningful taxa would be compared and eliminate most rare taxa. In NC, 7 bacterial taxa were significantly more abundant (*P* < 0.05), whereas 14 taxa were overrepresented in PC (*P* < 0.05) (Fig. 5a), and 2 bacterial taxa were significantly more abundant in NC (*P* < 0.05), whereas 38 taxa were overrepresented in FC (*P* < 0.05) (Figure S2a). No bacterial taxa were significantly more abundant in NC (*P* < 0.05), whereas only 2 taxa were overrepresented in BL (*P* < 0.05) (Fig. 5b). In BL, 2 bacterial taxa were significantly more abundant in BL (*P* < 0.05), whereas 10 taxa were overrepresented in PC (*P* < 0.05) (Figure S2b), and only one bacterial taxa was significantly more abundant in BL (*P* < 0.05), whereas 6 taxa were overrepresented in FC (*P* < 0.05) (Figure S2c). The results show that *Lactobacillus* and *Lactococcus* were significantly more abundant in NC than PC and FC (*P* < 0.05, Fig. 6a,b). However, *Bacteroides* and *Ruminococcus* were significantly more abundant in PC than in NC and BL (*P* < 0.05, Fig. 6c,d). The results also showed the abundance of *Streptomyces* and *Helicobacter* in four groups with a bar graph (Fig. 6e,f). The abundance of *Streptomyces* were more abundant in BL than in the other groups. In addition, the abundance of *Helicobacter* were more abundant in PC than in the other groups.

### Bacterial function prediction

**Figure 7:**
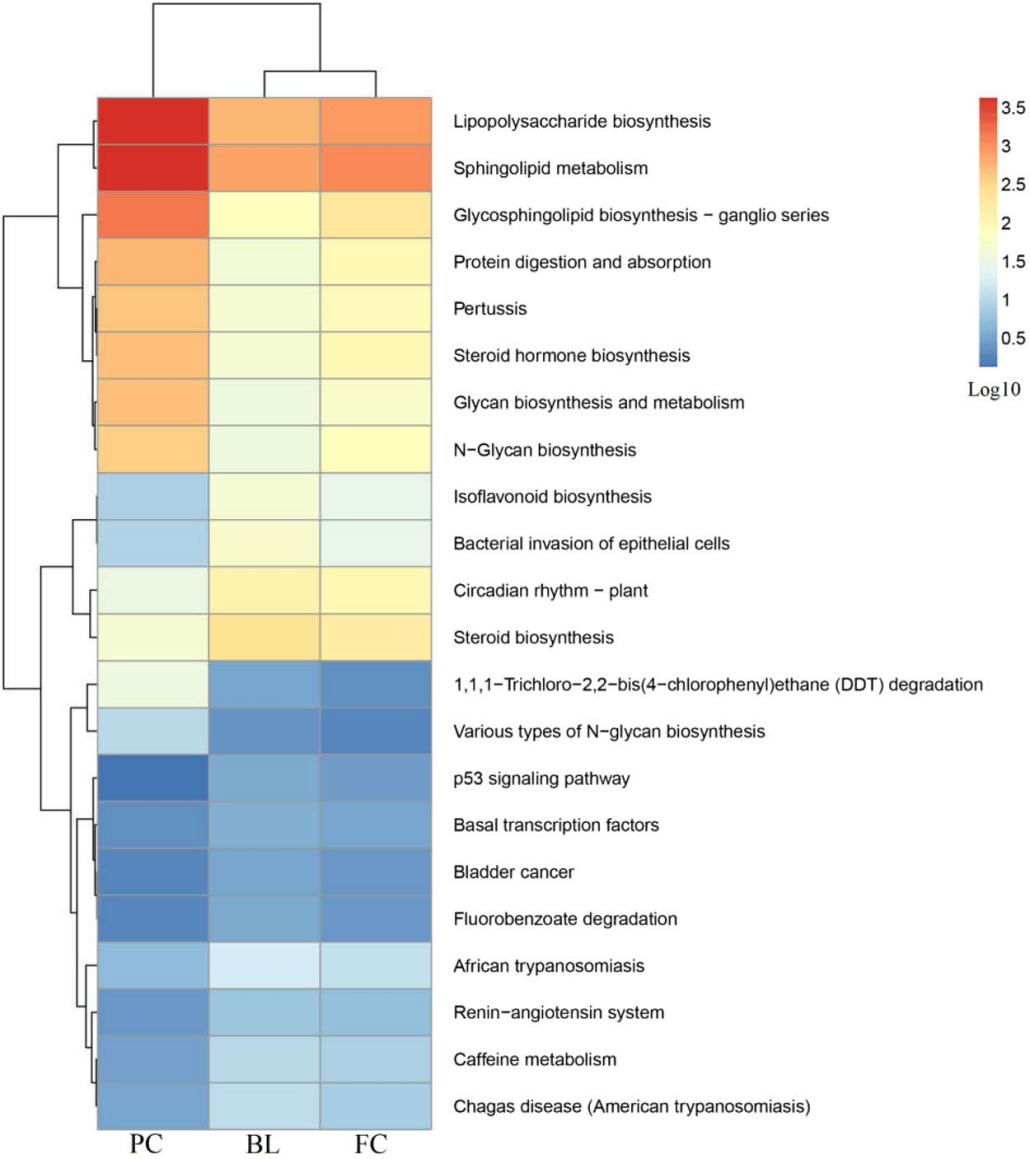
Predicted function in the gut micorbiota of FC, PC and BL. The gene copy numbers of samples within the same sample group were pooled. Values of each functional gene (row) were log10 transformed. The third level of the Kyoto Encyclopedia of Genes and Genomes pathway was shown in the heatmap. The significant test of the gene distribution between groups were performed using bootstrap Mann-Whitney u-test with cutoffs of *P* < 0.01, FDR < 0.1.

A computational tool, the Phylogenetic Investigation of Communities by Reconstruction of Unobserved States (PICRUSt), was used to predict the functional changes of the gut microbiota genomes in different treated groups from the Kyoto Encyclopedia of Genes and Genomes pathways. A total of 328 functions were detected in FC, PC and BL grounds, and 21 functions had a significant difference (bootstrap Mann-Whitney U-test, *P* < 0.01), which consisted of 6.40% of the overall functions, such as N−glycan biosynthesis, lipopolysaccharide biosynthesis and the P53 signalling pathway (Fig. 7).

## Discussion

Necrotic enteritis is a common disease of chickens. There are many predisposing factors of NE, but fishmeal and coccidium are the 2 most used factors. As reported, fishmeal feed can increase the amount of mucus secreted by the intestine and make the intestinal contents very viscous. Coccidia infection can directly damage the intestinal mucosa, induce the proliferation of pathogens and finally lead to NE infection. Weakened immunity also increase the probability that chickens are infected with NE^6^. The animal experiment of our published paper had used the same flocks of chickens, which found that the growth performance of the FC group was better than that of the PC group and none of the chickens in FC group were dead compared with the PC group^16^, which agreed with our previous study. Therefore, we speculated that chickens in the FC group were healthier than those of the PC group. This was because of the function of *C. perfringens*, which could produce toxins and enzymes such as α toxin^17^. This kind of toxin is a main A type toxin of *C. perfringens*, and it has the enzymatic activity of Phospholipase C and Sphingomyelinase. The alpha toxin can also hydrolyse Phosphatidylcholine and Sphingomyelin and destroy the cell membrane structure. It can induce the neutrophil granulocyte and macrophage to release TNF-α, which results in the haemolysis and death of the cell^18^. And that paper also showed that *B. licheniformis* supplement could improve the health status of chickens suffering from NE according to the results of mortality and growth performance^16^. The reason may be that *B. licheniformis* could enhance the gut barrier ability, which could stop the endotoxin and pathogens from passing through the intestinal mucosa into the blood. This can enhance the growth of immune organs, activate the lymphocyte, increase the level of immune globulin, and improve the cellular immunity and humoral immunity^19^. Simultaneously, *B. licheniformis* can also adjust the gut microbiota within animals and reduce the number of pathogens. It can also produce many bioactivators, such as lysozyme, bacteriocin, antifungal protein and varieties of antibiotics (including phospholipids, amino acids and polyenoid)^20^.

The gut microbiota community was relatively stable, which guarantees the healthy status of the host. The microbiota of the host plays an important role in the health of mankind by absorbing nutrients, enhancing growth and metabolism, protecting against harmful bacteria and modulating the immune system such that the function is irreplaceable. There have been studies showing that an intestinal flora disturbance could cause many diseases, and the health status of the same species has similar microbiota communities^21^. NE is a severe intestinal disease, and it can change the microbiota community structure^5^. We can observe that the gut microbiota communities can reflect the healthy status of the NE in chickens indirectly.

This study found that NC and BL were more similar than PC or FC in community richness and community diversity. The cause of the difference to NC may be explained by the damage of the ileum mucous membrane, which stimulates the growth of the invading organisms in PC and FC. For BL, *B. licheniformis* could probably prevent a flora disorder by mainly modulating mucosal immune activity and epithelial barrier function as a biological antagonist, which would reduce the succession of the invading organisms and indigenous microbiota. Using PCoA and gut microbiota tree analysis, we found that BL were more similar to NC and FC. This also might be because of the regulation by *B. licheniformis*.

Results of LEfSe showed that 21 bacterial taxa were significantly different between NC and PC, but only 2 bacterial taxa were significantly different between NC and BL. Therefore, we presume that NC and BL were more similar than PC in the ileum microbiota community.

Many gut microbiota species, especially *Lactobacillus*, *Lactococcus* and *Bacteroides*, have an intimate relationship with the health of animals. In addition, *Lactobacillus*, *Lactococcus* and *Bacteroides* were significantly different taxa based on the LEfSe analysis.

*Lactobacillus spp*., which is one of the most important bacteria in the intestinal tract of humans and animals, functions by maintaining the bacterial community in the intestinal tract, improving immunity, and facilitating the absorption of nutrients. Some studies show that *Lactobacillus* inhibited the inflammatory responses of the gut and had antagonistic effects against intestinal and food-borne pathogens by enhancing the immune functions in the intestinal mucosa^22^. *Lactobacillus* in the gut of animals can regulate the expression of the toll-like receptor gene, activate the immune response of DC/NK interaction, and balance the immune response of Th1/Th2^22^. In other words, the stability of *Lactobacillus* is very important to health. We found that the abundance of *Lactobacillus* in NC was significantly higher in PC but not significantly higher in BL. Therefore, we presume that BL may have stronger immune responses than PC. At the same time, we also found that the change in the abundances of *Lactobacillus* in chicken ileum were different than in chicken caecal pouches. Previous research showed that *Lactobacillus* of NE in chickens’ caecal pouches were changed in the composition of the species without changing the total abundance^5^. However, our study showed that the total abundances of *Lactobacillus* in PC and FC were significantly less than NC.

*Lactococcus spp*., a model LAB lactic acid bacteria, is one of the well-known lactate producing probiotics and is often used to improve the efficiency of animal digestion. The leading areas of study are the food-industry, biopharmaceuticals, and vaccines. The members of *Lactococcus* can produce bacteriocin (e.g., Nisin). Nisin is a member of the lantibiotic family of antimicrobial peptides that exhibit potent antibacterial activity against many gram-positive bacteria, including human and animal pathogens such as *Staphylococcus*, *Bacillus*, *Listeria*, and *Clostridium*^23^. *Lactococcus lactis*, which is a member of *Lactococcus spp*., can reduce the bacterial abundance of breeding ground for corruption, especially for *Leuconostoc*^24^. It is very important that the abundance of *Lactococcus* remain relatively stable. The results showed that the abundance of *Lactococcus* in BL were not significantly different from NC.

The LEfSe results showed that *Bacteroides spp*. were over-represented in PC. *Bacteroides* is a normal intestinal flora but can evolve into a pathogenic form, and the amounts could increase when the gut is pathologically changed or impaired^25^. Previous research shows that a member of *Bacteroides* could produce cell surface molecules. Cell surface molecules produced by this organism likely play important roles in colonization, communication with other microbes, and pathogenicity^26^, or even induce carcinoma of the intestine^27^. Human intestinal disease study analysis demonstrated that *Bacteroides* could cause diseases such as intra-abdominal infections^28^ and anaerobic bacteremia^29^. It could also enhance the resistance to antimicrobial agents such as antibiotics^30,31^ (e.g., carbapenems). The reason why *Bacteroides* were highest in PC may be explained by the fact that the disease of PC was the most serious. In BL, *Bacteroides* did not change, which may be due to the *B. licheniformis*, i.e., *B. licheniformis* can adjust the imbalance of normal intestinal flora and have a protective effect on the intestine.

*Ruminococcus* was the natural flora from the gut of chickens. In human studies, some *Ruminococcus* flora help cells to absorb sugars, which might contribute to weight gain^32^. The study showed that *Ruminococcus* in PC was higher than that of other groups. The relative results are the same as Willing *et al*.^33^ and Joossen *et al*.^34^, who studied on changes of intestinal microbiota of enteritis host, and the highly significant increase of the abundance of *Ruminococcus* was related closely to enteritis. There was another interesting report of increasing *Ruminococcus* in enteritis patients. It has been reported previously that *Ruminococcus* could produce lantibiotics, which could enhance sterilization activity against some clostridia and bifidobacteria species^35^. Thus, it was not desirable that the abundance of the *Ruminococcus* of the chicken ileum significant increased. Previous research showed that the increase in the number of *Ruminococcus* was related to mucin glycans^36^. Mucin, which is secreted by the gut epithelial cell, plays a key role in the gastrointestinal mucosal barrier, and it usually provides many adherens junctions and dietary requirements for bacteria. Some research showed its alterations were associated with numerous diseases, including carcinomas and inflammation^37^. The results of our study may be explained by the fact that the chicken ileum physiological environment was damaged, and the increased mucin was caused by damage, which finally led to an increasing abundance of *Ruminococcus*.

We found that the abundance of *Streptomyces* in BL was higher than in other groups. It also had an obvious dissimilarity between BL and the other groups, although LEfSe was not shown. *Streptomyces spp*., which have complex and large secondary metabolism regulatory networks, can produce many beneficial bioactive substances through secondary metabolism. *Streptomycin*, one of secondary metabolites of *Streptomyces*, has powerful antibacterial activity against mycobacterium tuberculosis and antimicrobial efficiency against a number of gram-negative bacteria (e.g., *Escherichia coli*, *Hepatitis bacillus*, *Enterobacter*, *Salmonella* and *Brucella*). In addition to antibiotics, the secondary metabolism of *Streptomyces* is varied, including anti-tumour agents, immune inhibitors, insect resistance agents and exocellular enzymes^38^ (e.g., pectinase, cellulase, and chitinase). In addition, recent research suggests that *Streptomyces spp*. could have benefits that extend beyond the gut, such as curing salmonella pullorum disease and *Pasteurella multocida* in infected chickens, but there are few studies on treating NE in chickens. It is also an anti-coccidial drug used in the poultry industry^39^, which is an important aetiological factor. Therefore, even *Streptomyces spp*. poorly exist in the chicken ileum by LEfSe, and it might be interesting to study the effects of *Streptomyces* in broiler chickens challenged with *C. perfringens*-induced NE.

We also found that a part of the different bacterial taxa were shared between the NC group and the other 2 groups (PC and FC), such as *Lactobacillus*. *Lactobacillus* was significantly more abundant in the NC group than the PC and FC groups in the LEfSe analysis, and the abundance of *Lactobacillus* in PC was less than FC. This may be related to the worsening of the illness. We found an interesting result, which showed that although chickens in the FC and PC group were infected with necrotic enteritis, *Lactococcus* was significantly more abundant in the FC group than the NC group, but it was significantly less in the PC group compared with the NC group. The healthy gut microbiota maintains relative stability or keeps the dynamic equilibrium status. Significantly increasing or decreasing the gut microbiota is bad for the host, especially the high relative abundance of a microorganism such as *Lactococcus*. This result might be similar to the impact on the host of changing the abundance of *Bacteroides*^25^.

The gut microbiota community was one of the factors affecting normal physiological functions. In human studies, abnormalities of the gut microbiota community can lead to allergies, obesity, diabetes and even cirrhosis of the liver^40,41^. The PICRUSt aims to predict the unobserved character states in a community of organisms from phylogenetic information regarding the organisms in the community. And they are commonly used to study animal intestinal function^42^. With respect to PICRUSt functional profiles, the enrichment of “Glycan Biosynthesis and Metabolism” (e.g., N−Glycan biosynthesis and lipopolysaccharide biosynthesis) pathways in the PC group is remarkable. This result was construable due to its high *Ruminococcus* abundance^32^. This was similar to our results of *Ruminococcus* abundance. We found that the P53 signalling pathway in the BL group is highly enriched. P53 is recognized as one of the important genes that participates in apoptosis control and is closely related to the incidence of a variety of human tumuors^43^. Inactivation of p53 by mutation occurs in over 50% of all human cancers. Some studies have shown that p53 inactivation is related to *Helicobacter pylor* infection^44^. Our study showed that the abundance of *Helicobacter* in PC was higher than the other groups. This was why the p53 signalling pathway in PC is not enriched. Chickens in PC was the most likely to develop bowel cancer, beacuse of the decrease of p53.

Our previous research showed that the supplement of *B. licheniformis* could significantly enhance the growth of immune organs^45^, number of white blood cells, mRNA expression levels of cytokines related to enteritis by the real-time PCR and villous histology of ileum was similar with those of the healthy chickens compared with chickens infected with NE^46^. The recent publication of ours also showed dietary *B. licheniformis* supplementation could adjust the expression levels of certain key genes related to lipid metabolism^3^. Our former paper also showed that probiotics could prevent NE by enhancing the immune function of ileum^47,48^. As everyone knows, gut microbiome can affect the immune function of host, the reason of which might be microbiome of ileum turns to be more balanced after probiotics treatment. At the same time, chickens of the BL group effectively alleviate the negative effects of NE infection and can also reduce antioxidant stress and enhance growth performance^5^. Therefore, we presume that chickens of the BL group was more health and hold more stable intestinal flora than chickens of the PC group.

In summary, the results of our study showed that the ileum microbiota of necrotic enteritis in chickens was normalized by dietary *B. licheniformis* supplementation. Furthermore, the data of this study may provide a new insight into the prevention and treatment of NE in broilers.

## Materials and Methods

### Test strain information

*B. licheniformis* H2 (CCTCC NO: M2011133) that was isolated from the ileum of healthy chickens was provided by the Animal Microecological Research Center (College of Veterinary Medicine, Sichuan Agricultural University, Chengdu, China).

The *C. perfringens* type-A (CVCC2030) strain isolated from a chicken clinically diagnosed with NE was obtained from the China Veterinary Culture Collection Center.

The DLV coccidium vaccine was developed by Shanghai Veterinary Research Institute, Chinese Academy of Agricultural Sciences.

### Experimental design and sampling

The experiments were approved before animal testing by the Sichuan Agricultural University Committee on Ethics in the Care and Use of Laboratory Animals. According to the Administration of Affairs Concerning Experimental Animals standard, the animals were subject to a carefully managed plan. The flowchart of this study is shown in Figure S3. Simply stated, 240 chickens were born on the same day with similar body masses and were purchased from a local commercial hatchery. All the samples were randomly assigned to four groups, five replicates per treatment, and twelve chickens per replicate in a pen. The four groups were (1) a negative control group fed with a corn-soybean meal diet (NC, negative control); (2) an NE experimental model group (PC, positive control); (3) a group that was fed a diet supplemented with 30% of fishmeal from day 14 onwards and challenged with the DLV coccidiosis vaccine (FC, fishmeal and coccidia); and (4) an infected group given a diet supplemented with *B. licheniformis* (BL, *B. licheniformis* at a dose of 1.0 × 10^6^ CFU/g). During this experiment, all the chickens were fed with free access to water and food in a temperature controlled room, and light was provided 24 hours a day. Table S2 shows the compositions of an un-medicated corn-soybean meal diet and a high fishmeal diet. The diets were formulated according to NRC (1994) (NRC. Nutrient Requirements of Poultry. 9th ed. Washington: The National Academies Press; 1994).

The birds were fed with a basal diet from day 1 to day 13. From day 14 onward, the diets of all the birds were changed to the basal diets supplemented with 30% fishmeal (w/w), except for those of the NC group. On day 15, all the birds, with the exception of those in the NC group, were inoculated with a 10-fold coccidiosis vaccine by oral gavage. In contrast, the birds in the NC group received sterile phosphate buffered saline. On days 18, 19, and 20, the birds in the PC and BL groups were individually infected with 1 mL of *C. perfringens* through a plastic tube containing approximately 2.2 × 10^8^ CFU/mL. The feed of the BL group was dosed with 1.0 × 10^6^ CFU/g *B. licheniformis* throughout the experiment.

On day 22, three chickens per pen were sacrificed randomly, and the contents in the ileum were collected. Sixty samples were immediately frozen in liquid nitrogen containers. The samples were then stored at −80 °C for further analysis.

All the experiments were performed in accordance with the approved guidelines and regulations.

### DNA extraction and pyrosequencing

We extracted the total bacteria DNA from the contents in the ileum (100 ± 10 mg each) by the EZNA Stool DNA Kit(Omega Bio-tek) according to the manufacturer’s instructions, and DNA samples were stored at −80 °C before further analysis. The sequencing was performed at the Chengdu Institute of Biology, Chinese Academy of Sciences. Basically, 16S rRNA genes of 16sV4 were amplified using 515 F/806 R (515 F: 5′-GTGCCAGCMGCCGCGGTAA-3′, 806 R: 5′-GGACTACVSGGG TATCTAAT-3′) with the barcode. The PCR amplification system is 100 μL, including 0.75 units Ex Taq DNA polymerase (TaKaRa, Dalian, China), 1 × Ex Taq loading buffer (TaKaRa, Dalian, China), 0.2 mM dNTP mix (TaKaRa, Dalian, China), 0.2 µM of each primer, and 100 ng template DNA. The conditions of the PCR amplification were 95 °C for 3 min (1 cycle), 94 °C for 100 s/50 °C for 60 s/72 °C for 60 s (35 cycles), and a last step of 72 °C for 10 min. The PCR-amplification products were purified by 2% agarose gel electrophoresis, and the EZNA Gel Extraction Kit (Omega Bio-tek) was used for the recovery of DNA from the gels.

### Bioinformatics and statistical analysis

The sequence reads were merged using mothur v1.31.2^49^. The sequences were assembled using Qiime 1.8.0 after the chimeric sequences were removed by Usearch 7.0.1001 and based on the Unweighted Pair-group Method with Arithmetic Means algorithm^50^. Operational taxonomic units (OTUs) were generated at a 97% similarity threshold, and sequences less than two were removed from all samples. The microbiota diversity analysis included an Alpha diversity analysis (Observed species and Shannon index) and Jackknifed beta diversity (weighted Unifrac distances calculated with 10 times of subsampling) in different samples, and weighted Unifrac distances were visualized by PCoA. The gut microbiota trees were generated using the weighted Pair Group Method with an Arithmetic Mean algorithm based on the different distance metrics generated by Qiime. The significant difference test of alpha diversity (Mann-Whitney U-test) and significance test of beta diversity (two-sided Student’s t-test) were analysed using QIIME. The significant difference test of the phylum level was analysed using SPSS 19.0. Linear discriminant analysis coupled with effect size (LEfSe) was performed to identify the differential expression in the genus level between the groups of bacterial taxa and compared with the relative contents of the differential expression in the genus level of the bacterial taxa. The function of the ileum microbiota community was predicted using PICRUSt^51^. The R packages “Biom”, “Phyloseq”, and “Pheatmap” were used for the data analysis and plotting. The raw read sequences of our 60 samples have been deposited at the Sequence Read Archive of the National Center for Biotechnology Information, with the study accession number SRP128297.

## Electronic supplementary material

Supplementary Information
