# Supplementary material for: Bacillus licheniformis normalize the ileum microbiota of chickens infected with necrotic enteritis
Source: Sci Rep. 2018 Jan 29;8:1744. doi: 10.1038/s41598-018-20059-z (PMC5789067; doi:10.1038/s41598-018-20059-z)
Supplement: Supplementary file 1 — Supplementary Information [file 41598_2018_20059_MOESM1_ESM.pdf]

Bacillus licheniformis normalize the ileum microbiota of chickens infected with necrotic enteritis

Shuai Xu[12*], Yicen Lin[12*], Dong Zeng[12*], Mengjia Zhou[12], Yan Zeng[12], Hesong Wang[12], Yi Zhou[12], Hui Zhu[12], Kangcheng Pan[1], Bo Jing[1], Xueqin Ni[12]

[1]Animal Microecology Institute, College of Veterinary Medicine, Sichuan Agricultural University, SichuanProvince, Chengdu 611130, China.

[2]Key Laboratory of Animal Disease and Human Health of Sichuan Province, Sichuan Agricultural University, Sichuan Province, Chengdu 611130, China.

[*]These authors contributed equally to this work.

Corresponding and requests for materials should be addressed to X.N. (xueqinni@foxmail.com)

Table S1 OTU table summary

Sample ID	SampleType	Group	Sequence counts	Observations (OTU number)
407	PC	PC_1	27977	765
408	PC	PC_2	29856	587
409	PC	PC_3	41145	531
410	PC	PC_4	20061	565
411	PC	PC_5	28939	696
G412	PC	PC_6	13326	292
413	PC	PC_7	34381	516
414	PC	PC_8	30311	536
415	PC	PC_9	17015	776
416	PC	PC_10	21421	357
417	PC	PC_11	29826	463
418	PC	PC_12	32714	295
419	PC	PC_13	36841	562
420	PC	PC_14	44121	409
421	PC	PC_15	12739	279
437	BL	BL_1	33171	160
438	BL	BL_2	9834	336
439	BL	BL_3	15708	255
440	BL	BL_4	21599	365
441	BL	BL_5	21376	362
442	BL	BL_6	19569	260
443	BL	BL_7	23862	304
444	BL	BL_8	8413	172
445	BL	BL_9	30892	355
446	BL	BL_10	18191	296
447	BL	BL_11	27671	228
448	BL	BL_12	34291	241
449	BL	BL_13	27550	805
450	BL	BL_14	21208	524
451	BL	BL_15	53547	507
452	FC	FC_1	17849	563
453	FC	FC_2	23352	630
O554	FC	FC_3	24093	239

455	FC	FC_4	13271	557
456	FC	FC_5	11686	773
457	FC	FC_6	13462	335
458	FC	FC_7	19574	351
459	FC	FC_8	40911	662
460	FC	FC_9	20296	654
461	FC	FC_10	18382	480
462	FC	FC_11	11540	497
463	FC	FC_12	29549	402
464	FC	FC_13	21327	413
465	FC	FC_14	32130	534
466	FC	FC_15	19638	576
467	NC	NC_1	23409	639
468	NC	NC_2	26738	411
469	NC	NC_3	27124	361
470	NC	NC_4	24584	251
471	NC	NC_5	16650	164
472	NC	NC_6	32812	625
473	NC	NC_7	44460	207
474	NC	NC_8	9363	144
475	NC	NC_9	30907	354
476	NC	NC_10	41433	362
477	NC	NC_11	28542	298
478	NC	NC_12	39900	454
O555	NC	NC_13	26345	154
480	NC	NC_14	25264	334
481	NC	NC_15	30613	480

Table S2 Composition and nutrient levels of basal diet

Ingredient (g/kg)	Corn-soybean meal diet	High fishmeal diet
Corn	51.64	53.80
Soybean	39.60	7.44
Fishmeal	0.00	30.00
Colza oil	4.30	4.30
Dicalcium phosphate	1.85	1.85
Limestone	1.30	1.30
D,L-Met	0.20	0.20
Salt	0.40	0.40
Choline	0.18	0.18
Vitamin Premix[a]	0.03	0.03
Mineral Premix[b]	0.50	0.50
Nutrient Level[c]		
Crude protein	21.17	25.98
Me (MJ/kg)	14.16	14.31
Methionine	0.49	0.95
Calcium	1.07	2.11
Total phosphorous	0.71	1.35

Note: [a] Multivitamines provided the following Per kilogram of basal diet: VA 50000IU,VD_3 10 000 IU,VE 25 IU, VB_1 2 mg,VB_2 16 mg,VB_6 6 mg,VB_{12} 0.03 mg, VK 35 mg, Nicotinic 25 mg,VB_3 25 mg, Folic acid 0.5 mg. [b] Microelement premix provided the following per kilogram of basal diet: Fe(as ferrous sulfate) 80.00 mg, Cu(as copper sulfate)8.00 mg, Mn(as manganese sulfate)60.00 mg, Zn(as zinc sulfate)40.00 mg, Se(as sodium selenite)0.15 mg, I(as potassium iodate)0.35mg.

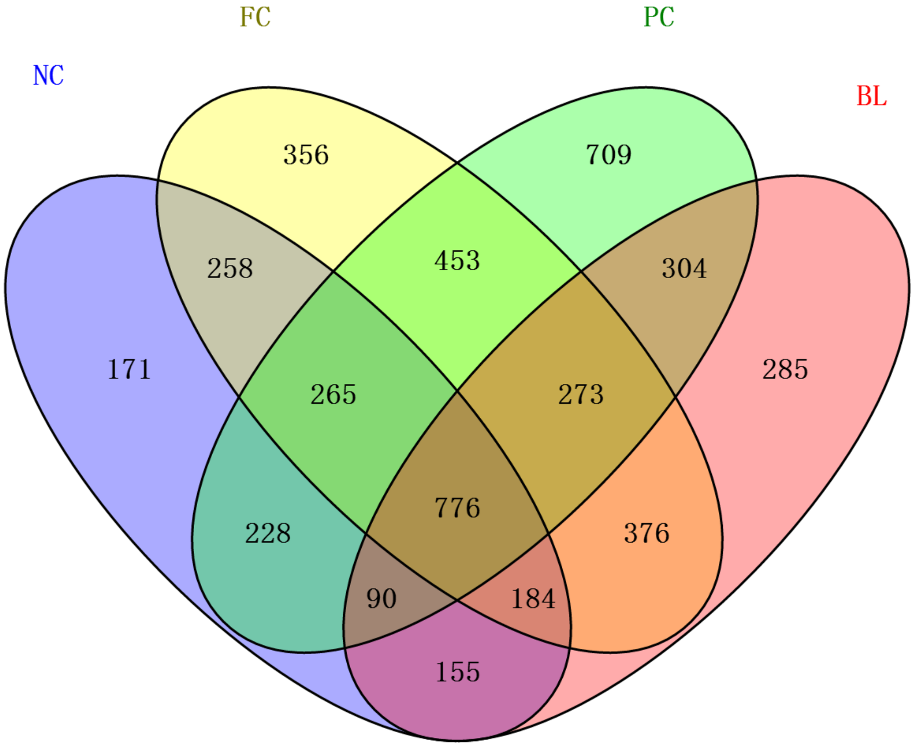

Figure S1 Shared operational taxonomic unit (OTU) analysis of the diferent communities.

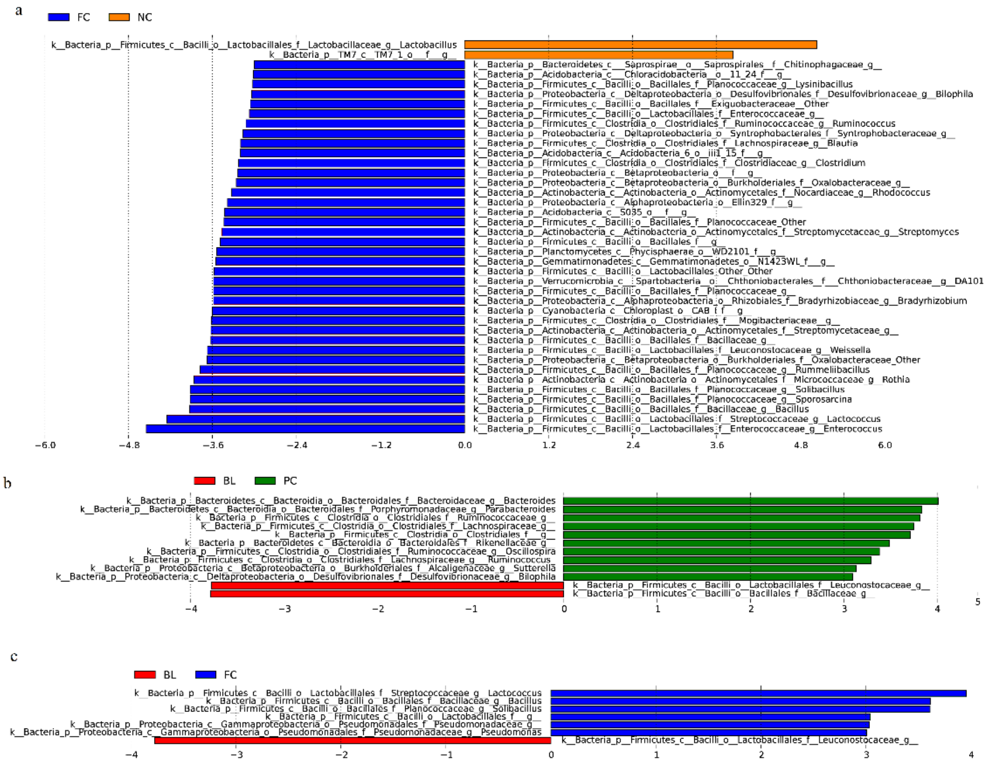

Figure S2 Taxa that were significantly differentially represented between groups were examined by linear discriminant analysis coupled with effect size (LEfSe) using the default parameters (LDA score = 3). Panel a show different taxa between NC and FC. Panel b show different taxa between BL and PC. Panel c show different taxa between BL and FC. The mean and median relative abundance of these bacterial taxa are indicated with straight and dotted lines, respectively.

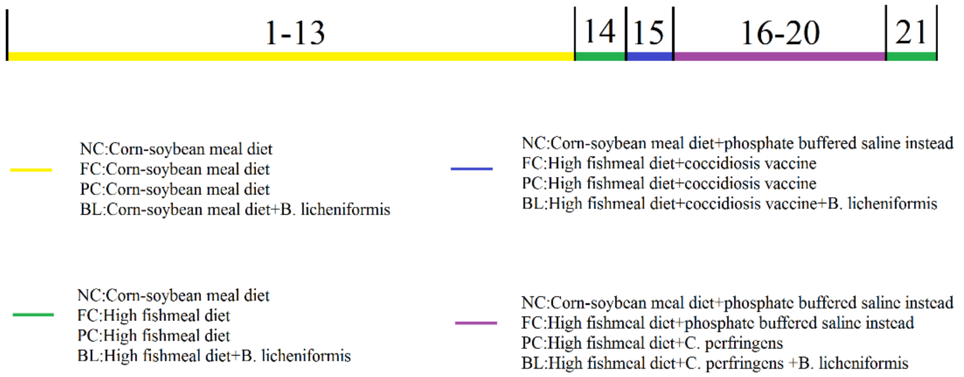

Figure S3 Flowchart of the experiment including breeding four groups.
